# Predicting Negative Emotions Based on Mobile Phone Usage Patterns: An Exploratory Study

**DOI:** 10.2196/resprot.5551

**Published:** 2016-08-10

**Authors:** Galen Chin-Lun Hung, Pei-Ching Yang, Chia-Chi Chang, Jung-Hsien Chiang, Ying-Yeh Chen

**Affiliations:** ^1^ Department of General Psychiatry Taipei City Psychiatric Center Taipei City Hospital Taipei Taiwan; ^2^ Department of Public Health, School of Medicine National Yang-Ming University Taipei Taiwan; ^3^ Department of Computer Science and Information Engineering National Cheng Kung University Tainan Taiwan; ^4^ Institute of Public Health and Department of Public Health National Yang-Ming University Taipei Taiwan

**Keywords:** mobile phone usage, depression, emotion, machine learning, affective computing

## Abstract

**Background:**

Prompt recognition and intervention of negative emotions is crucial for patients with depression. Mobile phones and mobile apps are suitable technologies that can be used to recognize negative emotions and intervene if necessary.

**Objective:**

Mobile phone usage patterns can be associated with concurrent emotional states. The objective of this study is to adapt machine-learning methods to analyze such patterns for the prediction of negative emotion.

**Methods:**

We developed an Android-based app to capture emotional states and mobile phone usage patterns, which included call logs (and use of apps). Visual analog scales (VASs) were used to report negative emotions in dimensions of depression, anxiety, and stress. In the system-training phase, participants were requested to tag their emotions for 14 consecutive days. Five feature-selection methods were used to determine individual usage patterns and four machine-learning methods were tested. Finally, rank product scoring was used to select the best combination to construct the prediction model. In the system evaluation phase, participants were then requested to verify the predicted negative emotions for at least 5 days.

**Results:**

Out of 40 enrolled healthy participants, we analyzed data from 28 participants, including 30% (9/28) women with a mean (SD) age of 29.2 (5.1) years with sufficient emotion tags. The combination of time slots of 2 hours, greedy forward selection, and Naïve Bayes method was chosen for the prediction model. We further validated the personalized models in 18 participants who performed at least 5 days of model evaluation. Overall, the predictive accuracy for negative emotions was 86.17%.

**Conclusion:**

We developed a system capable of predicting negative emotions based on mobile phone usage patterns. This system has potential for ecological momentary intervention (EMI) for depressive disorders by automatically recognizing negative emotions and providing people with preventive treatments before it escalates to clinical depression.

## Introduction

According to the World Health Organization, an estimated 350 million people are affected by depression worldwide. Depression is the leading cause of disability, and a major contributor to the global burden of disease, as measured by disability-adjusted life years [1]. Apart from pharmacological treatment and psychotherapy, self-management of negative emotions is of paramount importance because unprocessed negative emotions may escalate to clinical depression, and relapse of depression often results from an inadequate response to negative emotions [2]. In addition to those with clinical depression, the general public has developed an interest in recognizing their negative emotions, a major determinant of quality of life and adoption of health risk behaviors [3,4].

Awareness of negative emotion is crucial because it allows individuals to promptly respond with cognitive and behavioral strategies, avoiding escalation to clinical depression [5]. The concepts of ecological momentary assessment (EMA) and ecological momentary intervention (EMI) are the basis for real-time monitoring and management of context-specific emotional states [6,7]. A common and effective way to improve self-awareness is via regular self-tracking [8]. Several questionnaires pertaining to symptoms of depression have been implemented to mobile devices to help individuals track their emotional states [9-12].

In recent years, a novel and promising way for tracking emotion has emerged: via the mobile phone. The number of mobile phone users worldwide will surpass 2 billion in 2016 [13]. Mobile phone users spend almost an hour per day on their phones and check their phones on an average of 150 times daily [14]. Thus, mobile phones and mobile apps represent an opportunity to monitor and possibly intervene in mental health conditions [15,16]. In previous studies, mobile phones have shown potential for self-tracking of negative emotions [15,17,18].

### Automatic Detection of Emotion with Mobile Phone Sensors

Affective computing aims to automatically detect emotions when they arise [19,20]. Prior studies have attempted to infer emotions with various sensors. For example, the collection of voice and speech signals [21-26] has been attempted, but it requires the use of a camera or microphone, which is more invasive and power-consuming. Tracing physiological correlates of emotions, such as heart rate variability [27] and electro-dermal activity [28] via portable sensors is another viable strategy, though these parameters are often interfered by determinants other than emotion itself, including physical exertion and environmental conditions (eg, temperature, humidity [29]). Information retrieved from Global Positioning System (GPS) signals (geospatial activity), multi-axial accelerometers (kinesthetic activity), and light/sound sensors (ambient features, sleep) is frequently used to differentiate emotional statuses [16,18,23-26,30]. Samsung Technology also conducted studies related to emotion detection [31,32], which focused on users’ physical signals including hand gestures performed on the touch screen of a mobile phone.

### Using Mobile Phone Usage Patterns to Predict Emotional State

Behavioral patterns can either be an antecedent or a consequence of human emotion [33]. Mobile phone usage patterns are a traceable behavioral characteristic potentially associated with concordant emotional states [34]. Apps tracing mobile phone usage are generally lightweight and power efficient, without a need for computationally intensive or power-consuming data processing of video, audio or physiological signals [35]. Faurholt-Jepsen et al [24] demonstrated in patients with bipolar disorder possible correlations between number of calls and depressive symptoms. Saeb et al indicated that high phone usage was associated with depressive scores reported at baseline [30]. Burns et al [36] used mobile phone data, such as call logs, SMS (short message service) text messaging, and GPS to predict mood states, however, results were poor. Microsoft has developed the MoodScope system [37], which predicted users’ emotional state based on text messaging, emails, phone calls, application usage, Web browsing, and location and found that a personalized model with sufficient training period (ie, 2 months) would achieve a high predictive accuracy. However, few attempts have been made to compare the predictability of mobile phone usage data collected in different time frames (eg, 1 hour vs 2 hours prior to the reported emotion). In addition, a more sophisticated machine-learning algorithm was seldom applied.

The present study intends to capture user’s self-reported negative emotions and mobile phone usage data, and analyze their association in order to predict negative emotion. The specific aims of this study are (1) to develop a mobile phone application for data collection; (2) to use machine-learning methods for system building; and (3) to prospectively evaluate the system predictability.

## Methods

### Design

We developed a mobile app (iHOPE) to capture self-reported negative emotions and automatically collect mobile phone usage patterns in the background.

**Figure 1:**
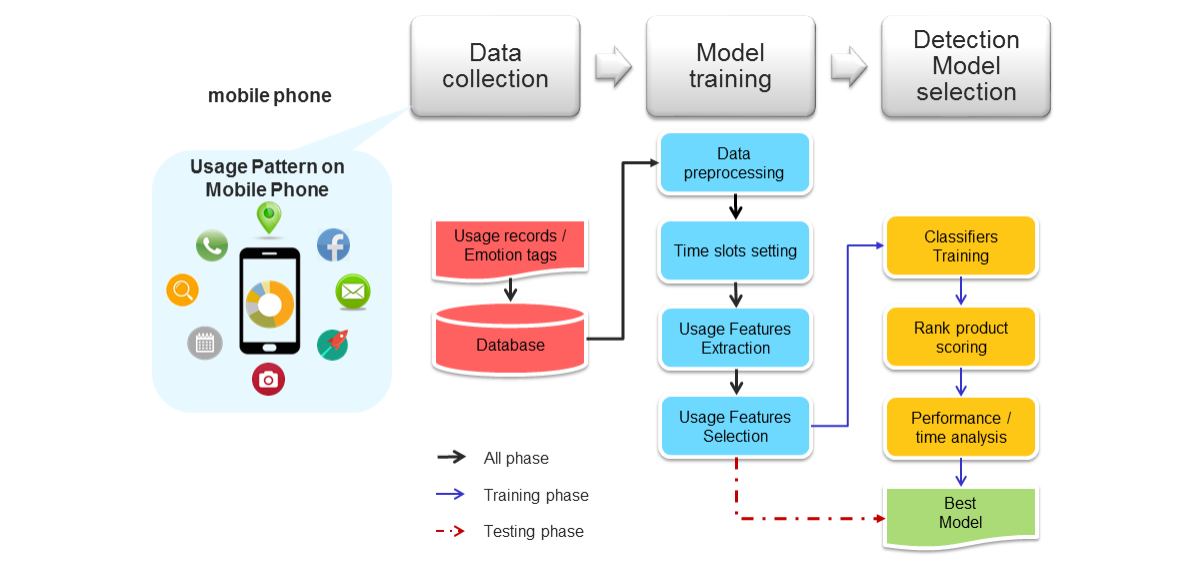
Study procedures.

The study procedure was divided into the following three phases (Figure 1): (1) data collection, which involves collecting negative emotion reports and raw mobile phone usage data, (2) classifier training, which involves quantifying negative emotions, defining usage patterns, feature extraction and selection, and selecting adequate time slots, with each combination being defined as a classifier, and (3) detection classifier selection where we developed a detection classifier selection method based on rank product scoring to reduce the number of combinations. We then evaluated the performance of each classifier and chose the best-performing one.

### Recruitment

**Figure 2:**
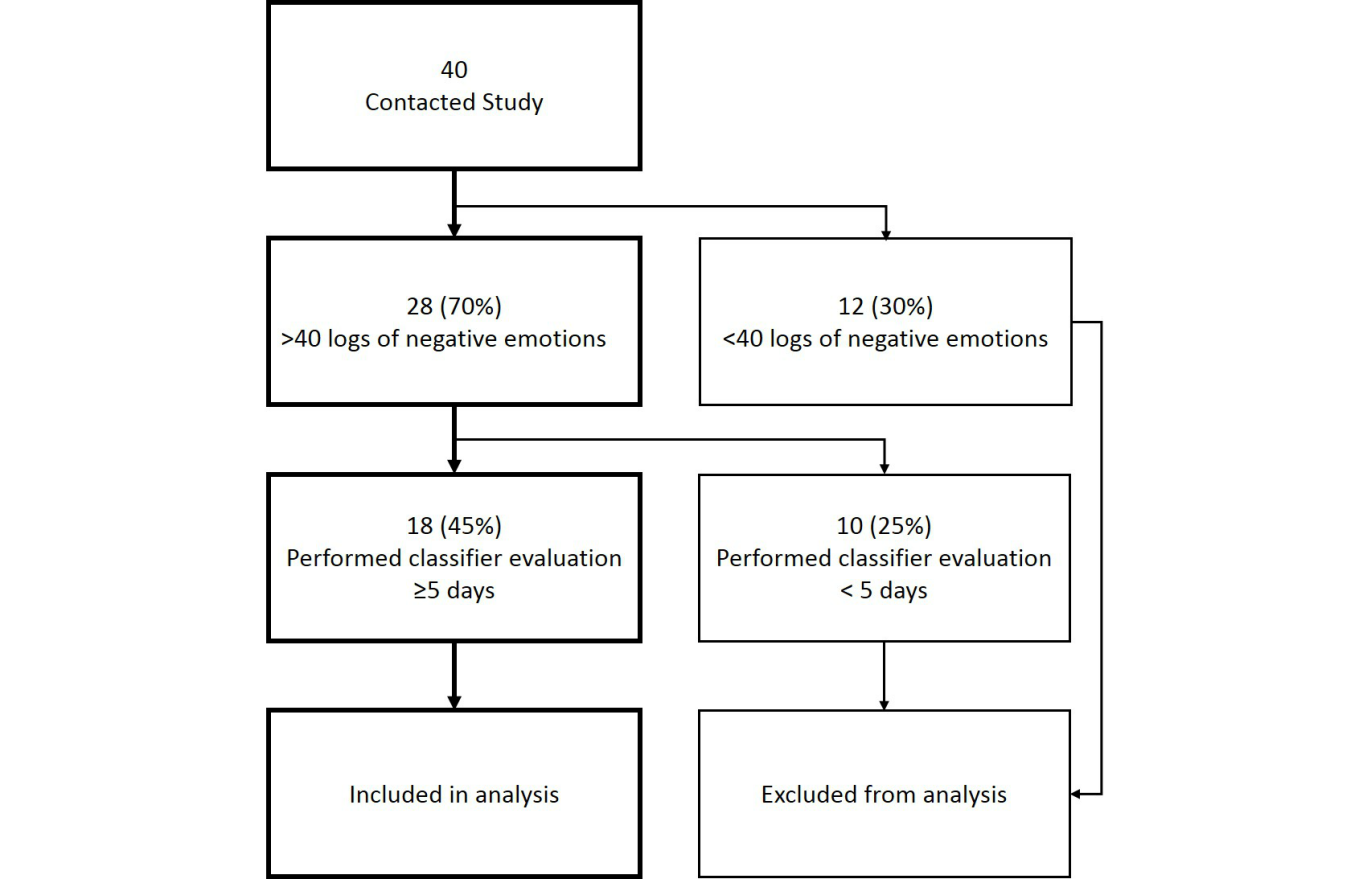
Study flow from recruitment to analysis.

Participants were graduate students recruited from the Institute of Medical Informatics, National Cheng Kung University, and medical professionals at Taipei City Psychiatric Center. In this exploratory study, we intended to recruit medical professionals with regular mobile phone use, better emotional awareness, and protocol adherence to properly train and validate our system. An invitation letter for participating in the study was sent to potential participants via email. After enrollment, they were requested to download and use the app on their primary mobile phone and tag the momentary state of depression, stress, and anxiety 4 times daily, with an interval of at least 3 hours between 2 consecutive tags, for 14 consecutive days. A notification was sent to the participants each time they were requested to tag emotions. All data were uploaded to a cloud server to train their personalized classifier. At the evaluation phase, we sent the personalized classifier via email for installation and requested the participants to validate the predicted negative emotions for at least 5 days. The study procedure is depicted in Figure 2.

### Data Collection

#### Negative Emotions

**Figure 3:**
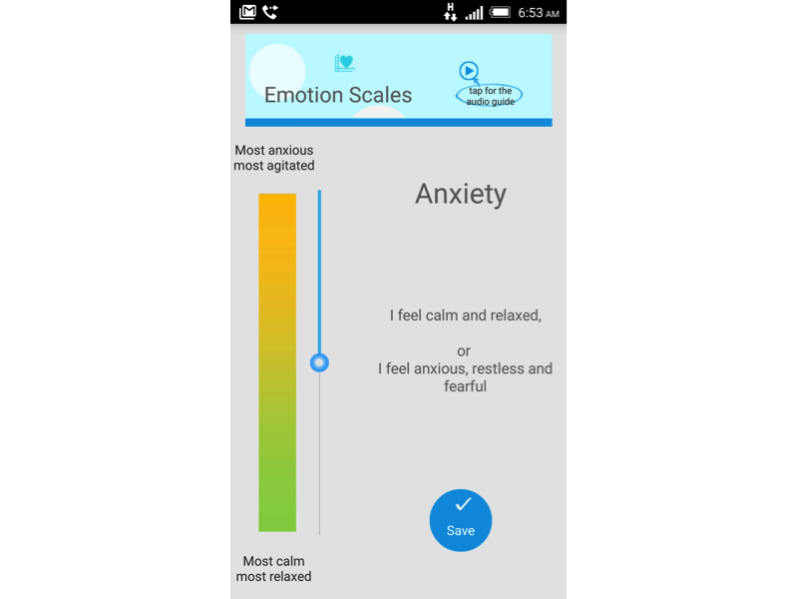
Visual analogue scale for anxiety.

In collaboration with a psychiatrist at Taipei City Psychiatric Center (GCH), we built the app with visual analogue scales (VASs) to measure negative emotions in three dimensions: depression, stress, and anxiety (Figure 3). Each VAS contained a slider for the user to drag to a specific point indicating their current level of emotion. For depression, the lowest level indicated normal mood, whereas the highest level reflected the most depressed state. For anxiety and stress, the lowest level represented the usual state, whereas the highest level indicated the most anxious and stressful condition.

#### Mobile Phone Usage Patterns

Our raw data contains call states and the package name of the app currently on the screen. We define the following three calling states: (1) idle state, the call function is not activated, (2) ringing state, which is a call waiting for the user to answer, with the phone either ringing or vibrating, and (3) off-hook state, that is, at least one call was dialing, active, or on hold. Our application monitored these calling states on the mobile phone every 3 seconds.

The package name of the app is recorded. To avoid duplicate app names, we record the package name of the app, which is unique in the installed-app list on the mobile phone. If there are several apps in the operating system hash, we only consider the app that currently occupies the screen. App categories, top ten apps, and screen usage are then inferred from the app package name records. Call-in, call-out, and missed calls are inferred from the call states.

### Timeslot Selection

We set a timeslot for every emotion tag, ranging from 0.5 hour, 1 hour, 1.5 hours, to 2 hours. In a given timeslot, the following mobile phone usage features were extracted: (1) count (the number of occurrences of the usage type within the timeslot), (2) total duration (the sum of the execution times), (3) average duration (the sum of the execution time divided by occurrence of usage), and (4) average interval (the average of the intervals between the start time of a usage and end time of previous usage of the same type). For example, if a time slot of 1 hour is selected, and we are determining the features of phone calls, we would extract the information regarding (during the hour preceding the emotional tag) the number of phone calls, the average and total durations of the phone calls, and the average duration of intervals between the two phone calls.

### Machine-Learning Methods Training

We used four conventional classifiers for analyzing mobile phone usage patterns (1) the Naïve Bayes classifier (NB); (2) the C4.5 decision tree (C4.5); (3) the Naïve Bayes decision tree (NBT); and (4) the Support Vector Machine (SVM) [38,39]. The classifiers are widely used because they are fast, easy to implement, and explain. The SVM generally performs the best, but it requires more complex processing and computing. The feature sets used to train the classifiers and the feature selection are personalized for the distinct features of negative emotions expressed by each participant.

**Figure 4:**
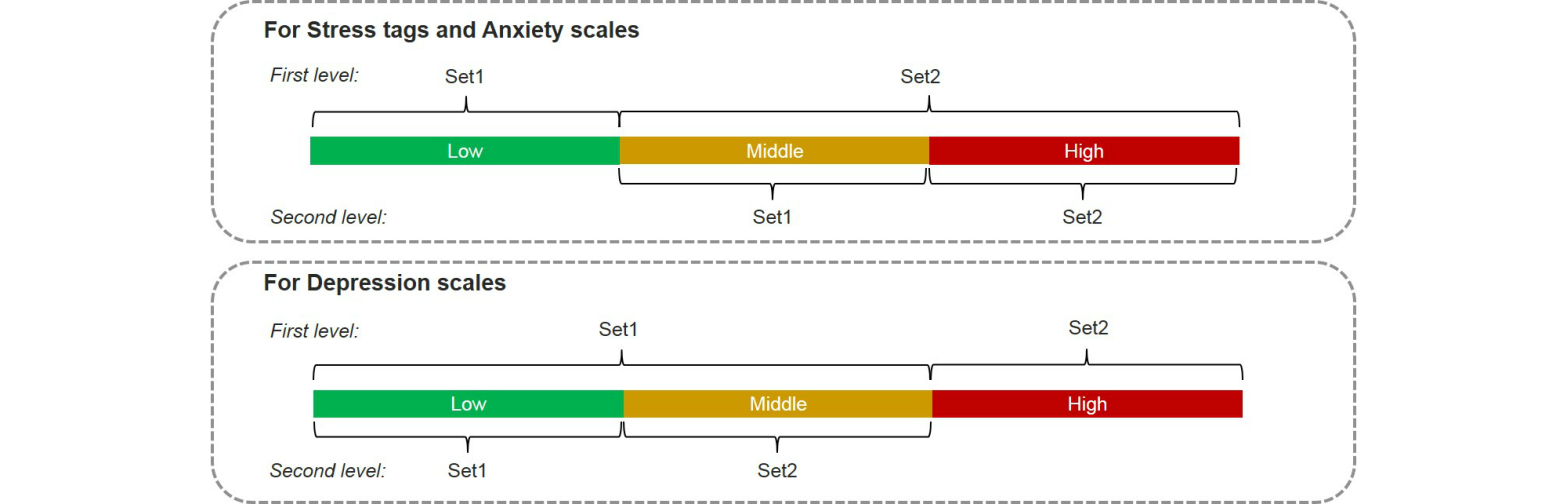
Two-level feature selection for scales of negative emotions.

Two types of 2-level *t* tests are applied for feature selection. The manner in which we grouped the sets for each level is illustrated in Figure 4. For the first 2-level *t* test selection, the features with *P* values less than .05 were chosen either in the first or second level. If less than five features were chosen, features with the smallest *P* values were selected. The *t* test was replaced by the homoscedastic *t* test for the other 2-level *t* test selection. This was different from the initial *t* test such that a smaller value represented greater similarity in the two testing sets. With the homoscedastic *t* test, all features were chosen in the beginning, and the features with *P* values less than .05 in the first or second levels of the test were deleted from the chosen set [40].

**Figure 5:**
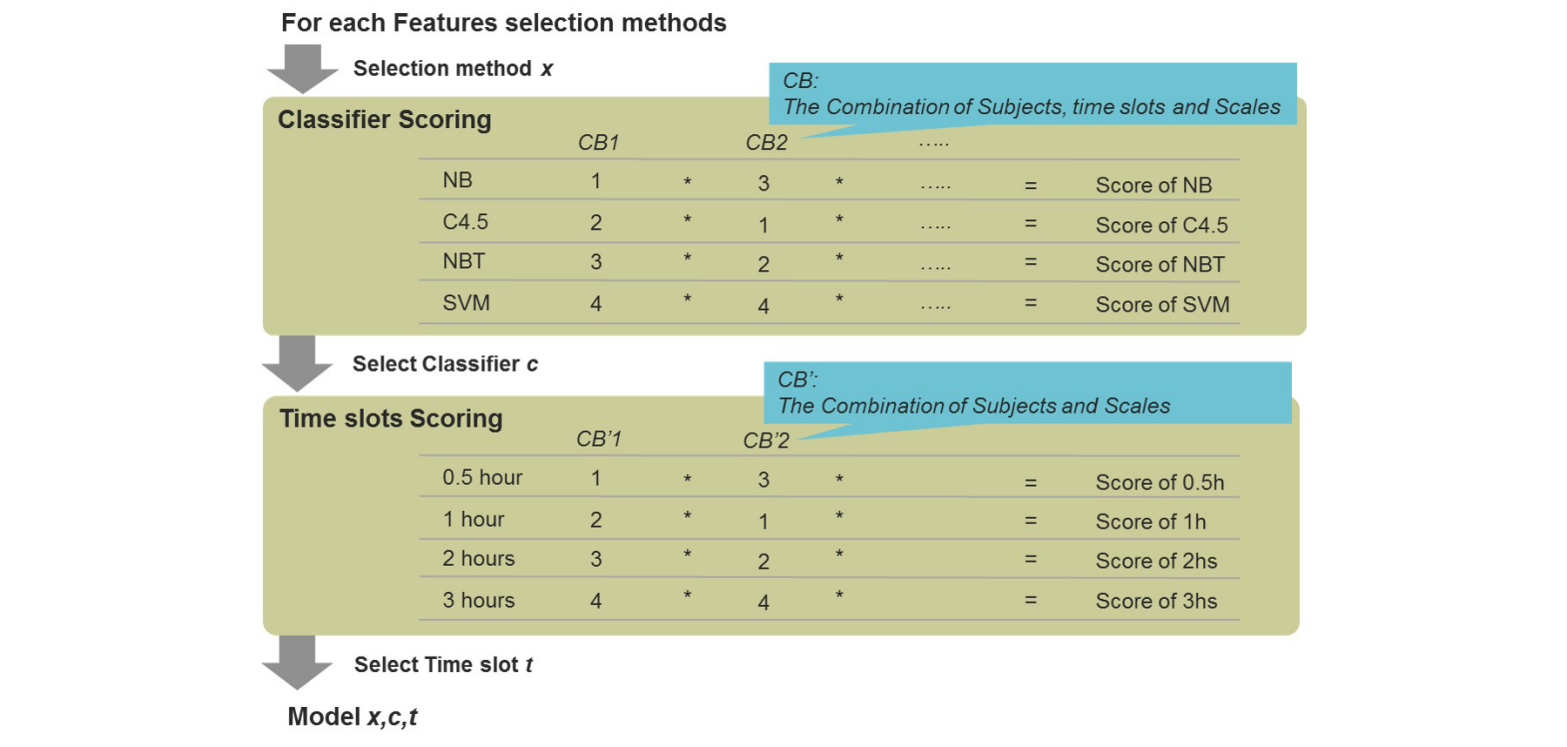
Detection classifier selection.

The best first feature selections used greedy algorithms to find the best feature set for classification instead of choosing each feature independently. These selection procedures were thus more likely to find multi-feature usage than the 2-level *t* test. We applied sequential forward selection, sequential backward selection, and bi-directional selection to do the best first selection. A detection classifier selection method was developed based on rank product scoring, which considered the rank order rather than the accuracy of the classification result, so that the influence of outliers was minimized. The process for detection classifier selection is depicted in Figure 5. After the combination for each feature selection method was determined, we selected the best classifier from the four classifiers based on the average predictive accuracy for the three VASs.

### Evaluation

After the personalized classifiers were trained using data collected from the 2-week period, it was provided to the participants for an evaluation for at least 5 days. Using the identical VAS for reporting negative emotions, the classifier provided predictions for current state of depression, anxiety, and stress every 2 hours from 11:00 AM to 11:00 PM. A notification was sent to the participants about the prediction, and they were allowed to correct it by dragging the slider to the exact level of current emotion. We divided the range of each negative emotion to three categories (low, medium, high). The prediction failed if any of the corrected ratings were not in the same category. We then compared the performance of our classifier with that of two benchmark models: multiple linear regression and general guess method.

## Results

### Study Sample

We recruited 40 healthy participants (30 graduate students and 10 medical professionals) who had contacted us to participate in the study. In the system-training phase, we used the data from 28 participants who had a minimum of 40 logs of negative emotions. Of them, 9 (32%, 9/28) were woman with a mean (SD) age of 29.2 (5.1) years. In the system-evaluation phase, we analyzed the data from 18 participants who had performed the evaluation for at least 5 days.

### Machine-Learning Methods Selection

To select the appropriate classifier, we tested four classifiers. The combination of the NB classifier with 2-hour timeslots and greedy best forward feature selection had the highest accuracy for the depression and stress scales. Thus, this combination was chosen as the best classifier to detect negative emotion and was applied to evaluate the individual classifier (refer to Multimedia Appendix 1 for a complete list of the performance achieved by every combination).

### Evaluation Outcomes

Among the 18 participants selected, they performed the evaluation for a mean (SD) of 10 (3) days. On average, an individual responded to 56 (20) predictions of their negative emotions. The results of the individual classifier evaluation are detailed in Table 1. In total, 1008 predictions were made and 995 of them (98.71 %, 995/1008) received feedback. Of those, 857 (86.1%, 857/995) indicated successful predictions, thus, the average rate of successful detections was 86.17%. Comparing with the predictive accuracy of multiple linear regressions (63%) and general guess method (77%), our classifier was substantially better in detecting negative emotions.

**Table 1:** Result of the personalized classifier evaluation.

User ID	Duration of system evaluation, days	Numbers of predictions responded^a^	Numbers of successful predictions	Predictive accuracy^b^, %
Subject 1	13	75	69	92.00
Subject 2	10	56	47	83.93
Subject 3	13	77	68	88.31
Subject 4	9	51	38	74.51
Subject 5	10	58	55	94.83
Subject 6	5	30	28	93.33
Subject 7	5	30	28	93.33
Subject 8	5	30	23	76.67
Subject 9	8	48	41	85.42
Subject 10	8	48	45	93.75
Subject 11	8	48	41	85.42
Subject 12	8	48	42	87.50
Subject 13	8	48	41	85.42
Subject 14	8	48	40	83.33
Subject 15	8	48	39	81.25
Subject 16	10	60	50	83.33
Subject 17	16	96	79	82.29
Subject 18	16	96	83	86.46
Total	10 (mean)	995	857	86.17

^a^The personalized classifier routinely made 7 predictions daily, but participants may respond only to some but not all of them.

^b^Successful predictions/responded predictions.

## Discussion

### Principal Findings

We developed a mobile phone app capable of reporting negative emotions and collecting mobile phone usage patterns. The present study provides preliminary evidence that by adapting sophisticated machine-learning methods, it is possible to predict concurrent negative emotions via mobile phone usage patterns with substantial accuracy.

### Limitations

The following limitations should be considered when interpreting the results of our pilot study. First, our participants were healthy professionals with moderate mobile phone usage and possibly better capability to define the degrees of negative emotions. A more extensive approach of validation would be recruiting a group of generally healthy controls, and another group with clinical depression. Second, mobile phone usage patterns is one but not the only indicator of underlying emotions. Other factors (eg, sleep, movement) are involved in manifesting the current emotional state and should be incorporated in future studies. Third, some participants either did not carry their mobile phones all the time or did not respond to emotion tags and predictions regularly, which interrupted data collection and prediction verification. Moreover, there were participants who used more than one mobile device. Therefore, data collected from a single mobile phone may not reflect the actual usage patterns. Lastly, users with higher suggestibility may be inclined to agree with the emotional state inferred. An alternative approach would be to ask users to report their momentary emotion before providing them with the prediction. Moreover, our system provided an individualized model for emotional prediction (ie, each participant had unique features of mobile phone usage patterns associated with her emotion). Due to the limited sample size, we were unable to identify any general patterns among the personalized features.

### Comparison with Previous Studies

The predictive accuracy of our system (86.17%) is comparative to previous research using mobile phone usage patterns to infer human emotions, however, the training time required (2 weeks) is substantially shorter than other studies (3 weeks to 3 months) [11,20,37]. We have observed that participants' usage behaviors changed substantially over time. Data currently collected for emotion inference may be out of the range of data previously used to obtain the personalized classifier, leading to failed prediction. Consequently, the personal classifier may need to be retrained periodically to improve the predictive accuracy. Aimed at preventing clinical depression, our predictions involve three negative emotions (depression, anxiety, and stress), which frequently precede a full-blown depressive episode, while prior studies [35,37] often adapted the circumplex model of normal emotion (ie, pleasure and activeness) [41]. While many studies used simple regression to predict emotion, we applied a sophisticated machine-learning process in combination with timeslot and feature selections. Moreover, when performing system evaluations, instead of adapting the same dataset used to train the personalized classifier, we request the users to prospectively validate the predicted emotion.

### Implications and Future Work

Though depressive disorders are becoming a global public health challenge, current treatment of depression has encountered considerable obstacles. A substantial portion of patients with depression either does not have access to standard treatments [1,42], primarily consisting of antidepressants and psychotherapy, or does not respond well to those treatments [43]. Outside of North America and Europe, there is a shortage of mental health professionals globally, and the stigma of mental disorders often prevents patients from actively seeking treatment [1]. Therefore, improving self-awareness and management of depression, or its antecedent negative emotion, can be a viable strategy for primary or secondary prevention. In addition, adopting the mobile phone interface can make it both accessible and scalable.

Analyzing mobile phone usage behavior to infer negative emotions is a pragmatic approach because it is possibly the single most prevalent, continuous, and traceable behavioral characteristic in the modern era. Future work should combine mobile phone usage patterns and other indices available for continuous monitoring via mobile phone(eg, location, weather) to collectively infer the underlying emotion. Moreover, emotion is a temporary state, which follows a dynamic pattern over time. A time-series analysis should be employed to account for the temporal alterations of negative emotions. With automatic detection of negative emotions, EMI becomes possible so that cognitive or behavioral suggestions may be provided seamlessly, and therefore bypassing the necessity of users having to acknowledge their negative emotions before receiving support. We are currently collaborating with the Taipei City Psychiatric Center, and are recruiting patients with depressive disorder to examine the feasibility and usefulness of this system in the clinical setting. The study protocol has been approved by the institutional review board (TCHIRB-1030206).

### Conclusions

In the present study, we developed a system capable of predicting negative emotions based on mobile phone usage patterns. The performance of our system appears to be superior to that of predictive models used in prior studies. The combination of timeslot selection, machine-learning process, and multiple feature selections may have substantially improved predictability. This system has a potential for ecological momentary intervention for health individuals or patients with depressive disorders by promptly recognizing negative emotion and providing them with preventive treatments before it escalates to clinical depression.

## Appendix

Multimedia Appendix 1 Comparison of performance achieved by different combinations of machine-learning methods.

## Abbreviations

EMI: ecological momentary intervention
GPS: Global Positioning System
NB: Naïve Bayes classifier
SMS: short message service
SVM: Support Vector Machine
VAS: visual analogue scale
