# Supplementary material for: Predicting Negative Emotions Based on Mobile Phone Usage Patterns: An Exploratory Study
Source: JMIR Res Protoc. 2016 Aug 10;5(3):e160. doi: 10.2196/resprot.5551 (PMC4997004; doi:10.2196/resprot.5551)
Supplement: Multimedia Appendix 1 [file resprot_v5i3e160_app1.pdf]

Appendix 1

Comparison of performance achieved by different combinations of machine-learning methods

We consider five feature selection methods (2 two-level T-Test selections and 3 greedy best first selections), four classifiers (NB, C4.5, NBT and SVM) and four sizes for the time windows (a half hour, 1 hour, two hours and 3 hours).

No.	Feature Selection Methods	Classifiers	Time Windows	Depression	Stress	Anxiety
1	All Features	NB	0.5 Hour	58.903	67.705	60.727
2	All Features	NB	1 Hour	59.598	71.681	64.235
3	All Features	NB	2 Hours	64.276	71.372	65.569
4	All Features	NB	3 Hours	63.027	70.442	68.825
5	All Features	J48	0.5 Hour	73.606	77.384	74.081
6	All Features	J48	1 Hour	68.651	77.861	70.949
7	All Features	J48	2 Hours	71.876	78.796	70.766
8	All Features	J48	3 Hours	71.311	75.003	70.445
9	All Features	NBTree	0.5 Hour	74.907	77.276	74.946
10	All Features	NBTree	1 Hour	70.136	76.991	76.282
11	All Features	NBTree	2 Hours	73.749	78.646	72.156
12	All Features	NBTree	3 Hours	71.022	79.067	76.407
13	All Features	SVM	0.5 Hour	68.165	78.025	72.687
14	All Features	SVM	1 Hour	67.265	73.849	70.321
15	All Features	SVM	2 Hours	71.765	79.512	74.484
16	All Features	SVM	3 Hours	68.802	77.534	74.161
17	T-Test Selection	NB	0.5 Hour	53.804	65.797	63.111
18	T-Test Selection	NB	1 Hour	59.098	63.283	59.505
19	T-Test Selection	NB	2 Hours	62.568	63.014	55.770
20	T-Test Selection	NB	3 Hours	64.851	65.672	60.678
21	T-Test Selection	J48	0.5 Hour	75.971	82.435	75.322
22	T-Test Selection	J48	1 Hour	73.439	80.318	72.916
23	T-Test Selection	J48	2 Hours	75.648	81.938	74.484
24	T-Test Selection	J48	3 Hours	74.977	81.432	77.312
25	T-Test Selection	NBTree	0.5 Hour	75.903	77.679	77.788
26	T-Test Selection	NBTree	1 Hour	74.674	77.599	78.437
27	T-Test Selection	NBTree	2 Hours	74.371	79.641	77.401
28	T-Test Selection	NBTree	3 Hours	77.069	80.303	77.290
29	T-Test Selection	SVM	0.5 Hour	75.202	82.016	80.154
30	T-Test Selection	SVM	1 Hour	76.657	82.179	79.322
31	T-Test Selection	SVM	2 Hours	78.491	81.978	77.609

No.	Feature Selection Methods	Classifiers	Time Windows	Depression	Stress	Anxiety
32	T-Test Selection	SVM	3 Hours	76.611	82.953	81.768
33	HT-Test Selection	NB	0.5 Hour	58.903	76.863	67.357
34	HT-Test Selection	NB	1 Hour	59.598	78.489	72.479
35	HT-Test Selection	NB	2 Hours	64.276	80.225	74.522
36	HT-Test Selection	NB	3 Hours	63.027	80.031	77.407
37	HT-Test Selection	J48	0.5 Hour	73.606	81.398	76.328
38	HT-Test Selection	J48	1 Hour	68.651	81.256	74.220
39	HT-Test Selection	J48	2 Hours	71.876	83.018	76.135
40	HT-Test Selection	J48	3 Hours	71.311	82.325	78.194
41	HT-Test Selection	NBTree	0.5 Hour	74.907	79.858	76.847
42	HT-Test Selection	NBTree	1 Hour	70.136	77.953	78.248
43	HT-Test Selection	NBTree	2 Hours	73.749	81.516	77.821
44	HT-Test Selection	NBTree	3 Hours	71.022	82.428	80.978
45	HT-Test Selection	SVM	0.5 Hour	68.165	82.500	77.361
46	HT-Test Selection	SVM	1 Hour	67.265	81.650	77.295
47	HT-Test Selection	SVM	2 Hours	71.765	84.219	78.527
48	HT-Test Selection	SVM	3 Hours	68.802	82.877	81.482
49	Greedy Forward Selection	NB	0.5 Hour	80.416	85.456	81.473
50	Greedy Forward Selection	NB	1 Hour	77.256	83.859	81.591
**51**	**Greedy Forward Selection**	**NB**	**2 Hours**	**81.985**	**84.580**	**82.965**
52	Greedy Forward Selection	NB	3 Hours	77.040	84.137	81.735
53	Greedy Forward Selection	J48	0.5 Hour	78.477	84.554	78.161
54	Greedy Forward Selection	J48	1 Hour	80.498	80.795	81.453
55	Greedy Forward Selection	J48	2 Hours	81.214	82.722	78.840
56	Greedy Forward Selection	J48	3 Hours	78.310	81.779	80.557
57	Greedy Forward Selection	NBTree	0.5 Hour	78.876	80.404	80.835
58	Greedy Forward Selection	NBTree	1 Hour	78.359	80.369	80.905
59	Greedy Forward Selection	NBTree	2 Hours	78.541	82.628	81.753
60	Greedy Forward Selection	NBTree	3 Hours	77.638	82.914	81.488
61	Greedy Forward Selection	SVM	0.5 Hour	76.545	80.216	78.008
62	Greedy Forward Selection	SVM	1 Hour	75.584	79.472	78.731
63	Greedy Forward Selection	SVM	2 Hours	78.512	80.022	78.999
64	Greedy Forward Selection	SVM	3 Hours	77.203	82.465	81.766
65	Greedy Backward Selection	NB	0.5 Hour	67.019	74.984	69.486
66	Greedy Backward Selection	NB	1 Hour	65.926	77.013	76.330
67	Greedy Backward Selection	NB	2 Hours	69.682	76.381	76.596
68	Greedy Backward Selection	NB	3 Hours	72.012	79.267	77.412
69	Greedy Backward Selection	J48	0.5 Hour	74.083	82.512	76.685

No.	Feature Selection Methods	Classifiers	Time Windows	Depression	Stress	Anxiety
70	Greedy Backward Selection	J48	1 Hour	75.529	80.044	77.749
71	Greedy Backward Selection	J48	2 Hours	76.333	80.664	79.949
72	Greedy Backward Selection	J48	3 Hours	77.041	80.698	78.299
73	Greedy Backward Selection	NBTree	0.5 Hour	78.668	79.023	80.181
74	Greedy Backward Selection	NBTree	1 Hour	76.323	80.201	81.231
75	Greedy Backward Selection	NBTree	2 Hours	79.668	81.862	81.411
76	Greedy Backward Selection	NBTree	3 Hours	78.752	82.671	80.721
77	Greedy Backward Selection	SVM	0.5 Hour	76.094	78.853	77.781
78	Greedy Backward Selection	SVM	1 Hour	76.726	81.062	78.049
79	Greedy Backward Selection	SVM	2 Hours	76.849	80.166	79.320
80	Greedy Backward Selection	SVM	3 Hours	77.659	80.724	79.526
81	Greedy Bi-Directional Selection	NB	0.5 Hour	80.416	85.456	81.473
82	Greedy Bi-Directional Selection	NB	1 Hour	77.256	83.859	81.591
83	Greedy Bi-Directional Selection	NB	2 Hours	81.530	84.580	82.965
84	Greedy Bi-Directional Selection	NB	3 Hours	77.040	84.137	81.735
85	Greedy Bi-Directional Selection	J48	0.5 Hour	77.867	84.554	78.161
86	Greedy Bi-Directional Selection	J48	1 Hour	80.201	80.795	81.453
87	Greedy Bi-Directional Selection	J48	2 Hours	81.214	82.327	78.840
88	Greedy Bi-Directional Selection	J48	3 Hours	77.091	81.779	80.557
89	Greedy Bi-Directional Selection	NBTree	0.5 Hour	78.876	80.404	80.835
90	Greedy Bi-Directional Selection	NBTree	1 Hour	78.359	80.369	80.905
91	Greedy Bi-Directional Selection	NBTree	2 Hours	78.541	82.727	81.753
92	Greedy Bi-Directional Selection	NBTree	3 Hours	76.916	82.914	81.488
93	Greedy Bi-Directional Selection	SVM	0.5 Hour	76.545	80.216	78.008
94	Greedy Bi-Directional Selection	SVM	1 Hour	75.584	79.472	78.731
95	Greedy Bi-Directional Selection	SVM	2 Hours	78.512	79.923	78.999
96	Greedy Bi-Directional Selection	SVM	3 Hours	76.337	82.465	81.766
